# Supplementary material for: Ibrutinib reverses IL-6-induced osimertinib resistance through inhibition of Laminin α5/FAK signaling
Source: Commun Biol. 2022 Feb 23;5:155. doi: 10.1038/s42003-022-03111-7 (PMC8866396; doi:10.1038/s42003-022-03111-7)
Supplement: Supplementary file 2 — Supplementary Information [file 42003_2022_3111_MOESM2_ESM.pdf]

Supplementary Information for

Ibrutinib reverses IL-6-induced osimertinib resistance through inhibition of Laminin α5/FAK signaling

Authors

Li Li [1, †, *], Zhujun Li[1, †], Conghua Lu[1, †], Jianghua Li[1], Kejun Zhang[2], Caiyu Lin[1], Xiaolin Tang[1], Zhulin Liu[1], Yimin Zhang[1], Rui Han[1], Yubo Wang[1], Mingxia Feng[1], Yuan Zhuang[3], Chen Hu [1, *], Yong He[1, *]

Affiliations

[1] Department of Respiratory Medicine, Daping Hospital, Third Military Medical University (Army Medical University), Chongqing 400042, China

[2] Department of Clinical Laboratory, Daping Hospital, Third Military Medical University (Army Medical University), Chongqing 400042, China

[3] National Engineering Research Center of Immunological Products, Department of Microbiology and Biochemical Pharmacy, College of Pharmacy and Laboratory Medicine, Third Military Medical University (Army Medical University), Chongqing 400038, China

[†] These authors contributed equally to this work.

***Correspondence author:** Yong He, Department of Respiratory Medicine, Daping Hospital, Army Medical University, Chongqing 400042, China. Phone: 86-23-68729191; Fax: 86-23-68729191; E-mail: heyong@tmmu.edu.cn; or Chen Hu, huchen89@tmmu.edu.cn; Li Li, dpyyhxlili@tmmu.edu.cn

This PDF file includes:

Supplementary Figures 1-8
Supplementary Table 1-2

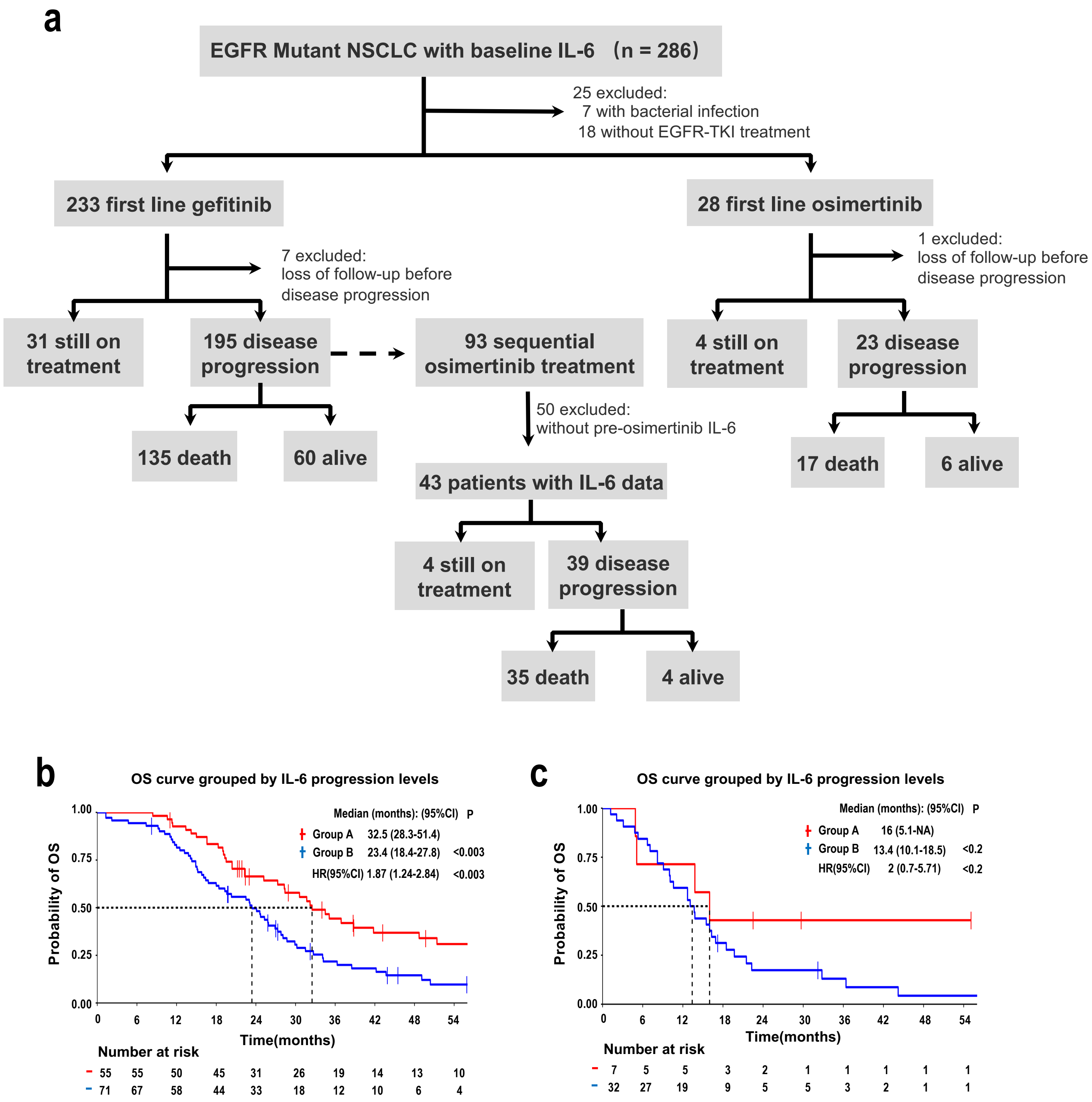

Supplementary Figure 1. Flow chart of the study population and OS analysis upon disease progression. (a), Study flow chart. (b) and (c), Kaplan–Meier (KM) estimates of OS in NSCLC patients from progression on gefitinib, or osimertinib, respectively, according to IL-6 dynamics. Group A: those with decreased or slightly increased IL-6 levels (value < 7mg/L upon disease progression); Group B: those with highly elevated IL-6 levels upon disease progression (value ≥ 7mg/L upon resistance and higher than that of baseline). OS, overall survival.

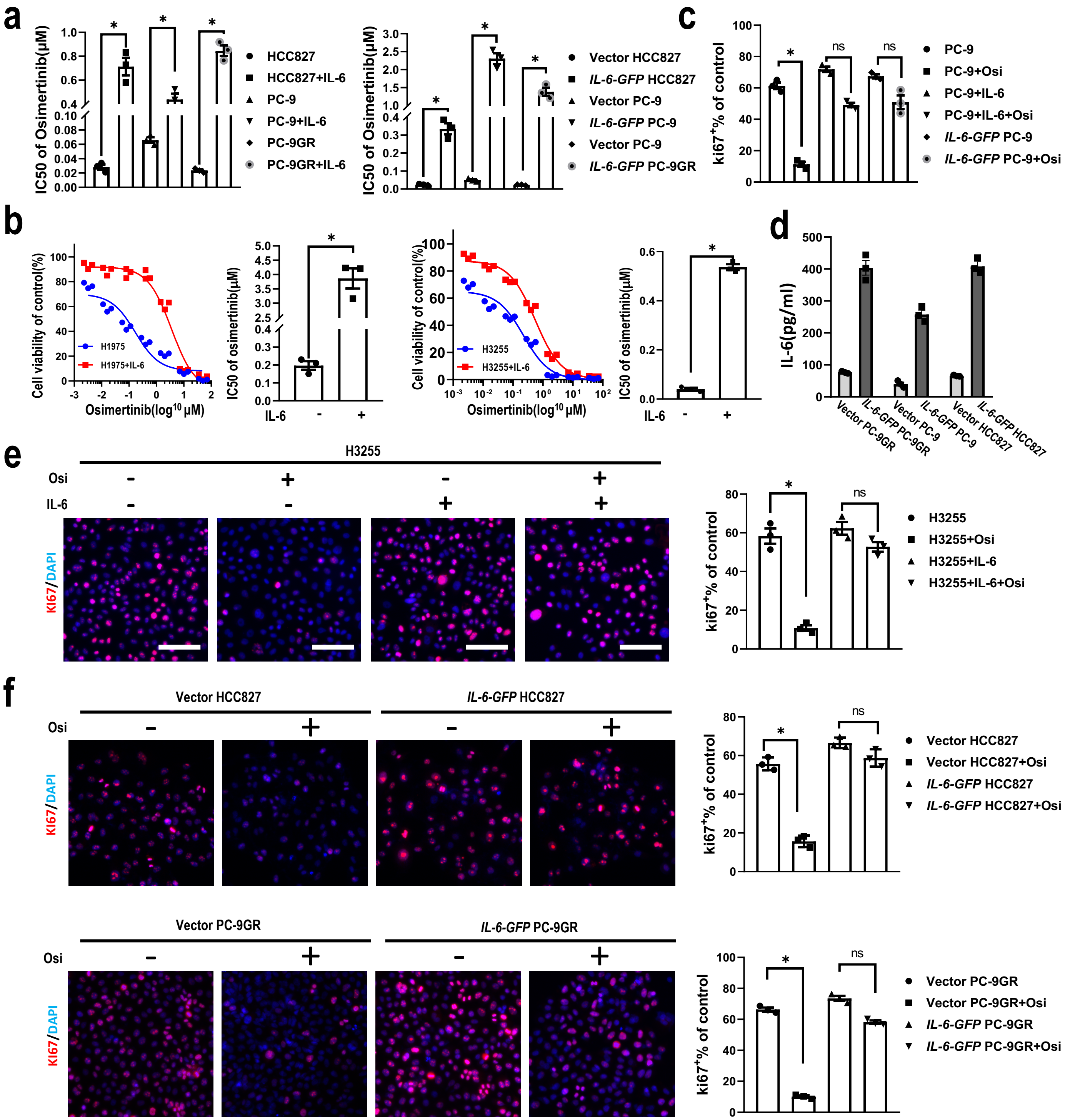

Supplementary Figure 2. IL-6 could induce osimertinib resistance in osimertinib-sensitive cells. (a), Histogram shows IC_{50} values in the indicated groups (Related to Figure 1H, *p < 0.01 by Student's t-test). (b), Cell viability CCK-8 assay for H1975 and H3255 cells treated with IL-6 (20ng/ml) together with increasing concentrations of osimertinib for 48h. (c), Histogram shows percentage of Ki67 values in the indicated groups (Related to Figure 1I, *p < 0.01 by Student's t-test). (d), ELISA analysis of IL-6 levels from culture medium of indicated cell lines. (e), Ki67 incorporation assay on H3255 cells treated with IL-6 (20ng/ml) together with osimertinib for 48h. (f), Ki67 incorporation assay on *IL-6-GFP* HCC827 cells and *IL-6-GFP* PC-9GR cells with different treatments as indicated. Osimertinib (1μM) were added to the culture medium for 48h. Cells were counterstained with DAPI. Histogram shows Ki67 positive percentages in different groups as indicated (*p < 0.001 by Student's t-test). (n=3 biologically independent experiments).

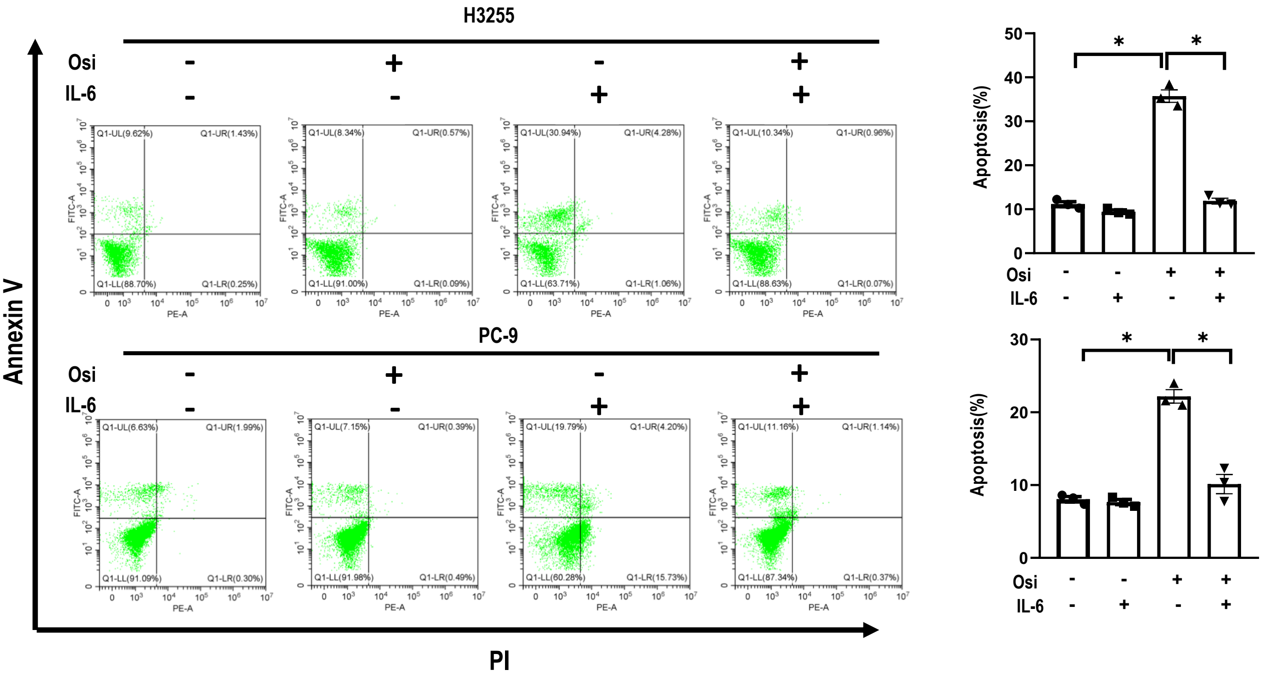

Supplementary Figure 3. Flow cytometry analysis of Annexin V and PI to detect apoptosis in H3255 and PC-9 cells with different treatments as indicated (n=3 biologically independent experiments). *$p < 0.01$ by Student's t-test

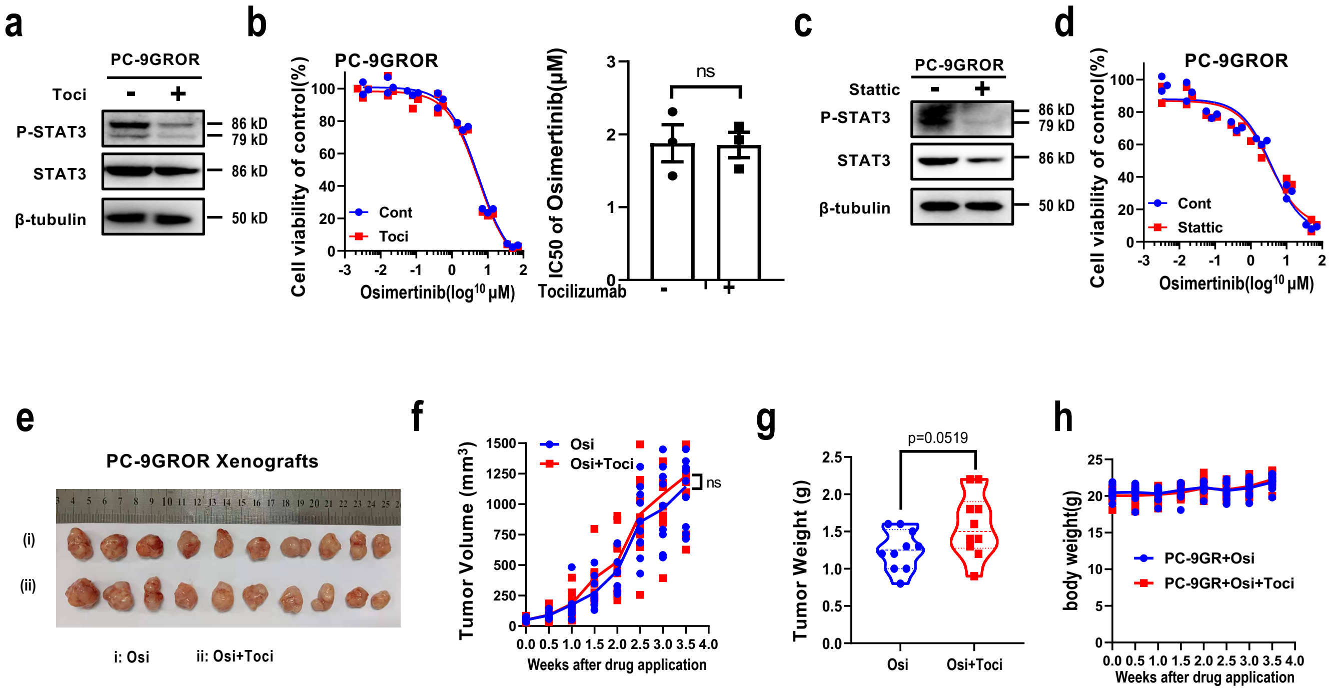

Supplementary Figure 4. **Tocilizumab or Stattic failed to reverse osimertinib resistance in vitro and in vivo**. (a), Western blot showing the expression levels of indicated proteins in PC-9GROR cells in the presence or absence of tocilizumab (5 µg/ml) for 48h. (b), Cell viability CCK-8 assay for PC9GROR cells treated with tocilizumab (5 µg/ml) together with increasing concentrations of osimertinib for 48h (n=3 biologically independent experiments). Histogram shows IC50 values in the indicated groups. (c), Western blot showing the expression levels of indicated proteins in PC-9GROR cells in the presence or absence of Stattic (10µM) for 48h. (d), Cell viability CCK-8 assay for PC-9GROR cells treated with Stattic (10µM) together with increasing concentrations of osimertinib for 48h. (e), Macroscopic appearance and tumor weights of the xenografts in different groups as indicated (n=10 biologically independent animals). (f), Tumor growth of PC-9GROR xenografts treated with osimertinib (20 mg/L), or the combination of osimertinib (20 mg/L) and tocilizumab (100 mg/mice, i.p.). Tumor sizes were presented as mean ± SEM .(g), Tumor weights of individual tumors from two groups. (h), mice body weight of the two groups following treatments.

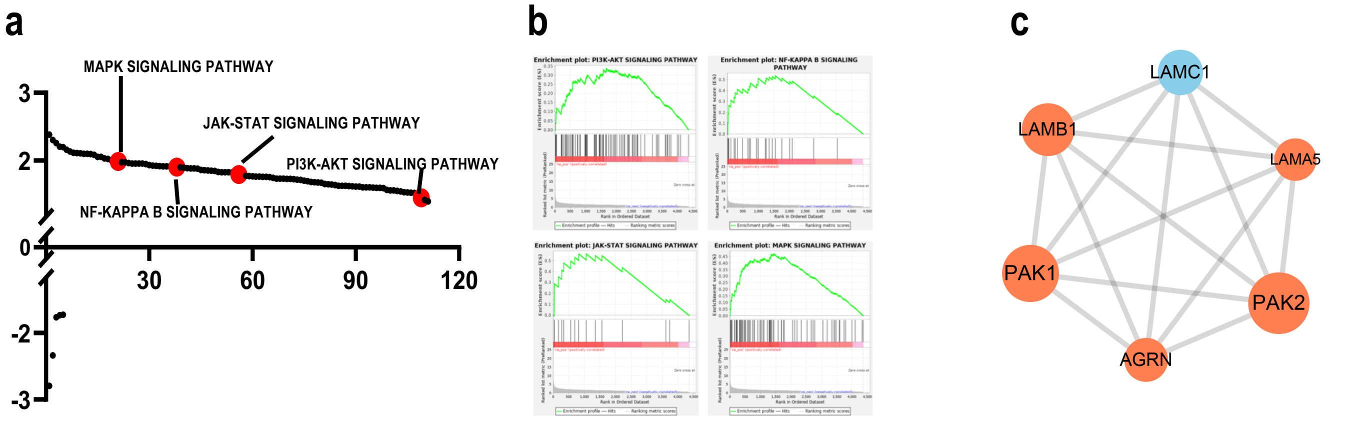

Supplementary Figure 5. Proteomic analysis (Related to Figure 2). (a), The distribution of enriched pathways by NES value. Red nodes refer to four selected signaling pathways downstream of IL-6. (b), Gene set enrichment analysis (GSEA) comparing proteins that changed after osimertinib resistance in the four indicated pathways. (c), the Focal adhesion category

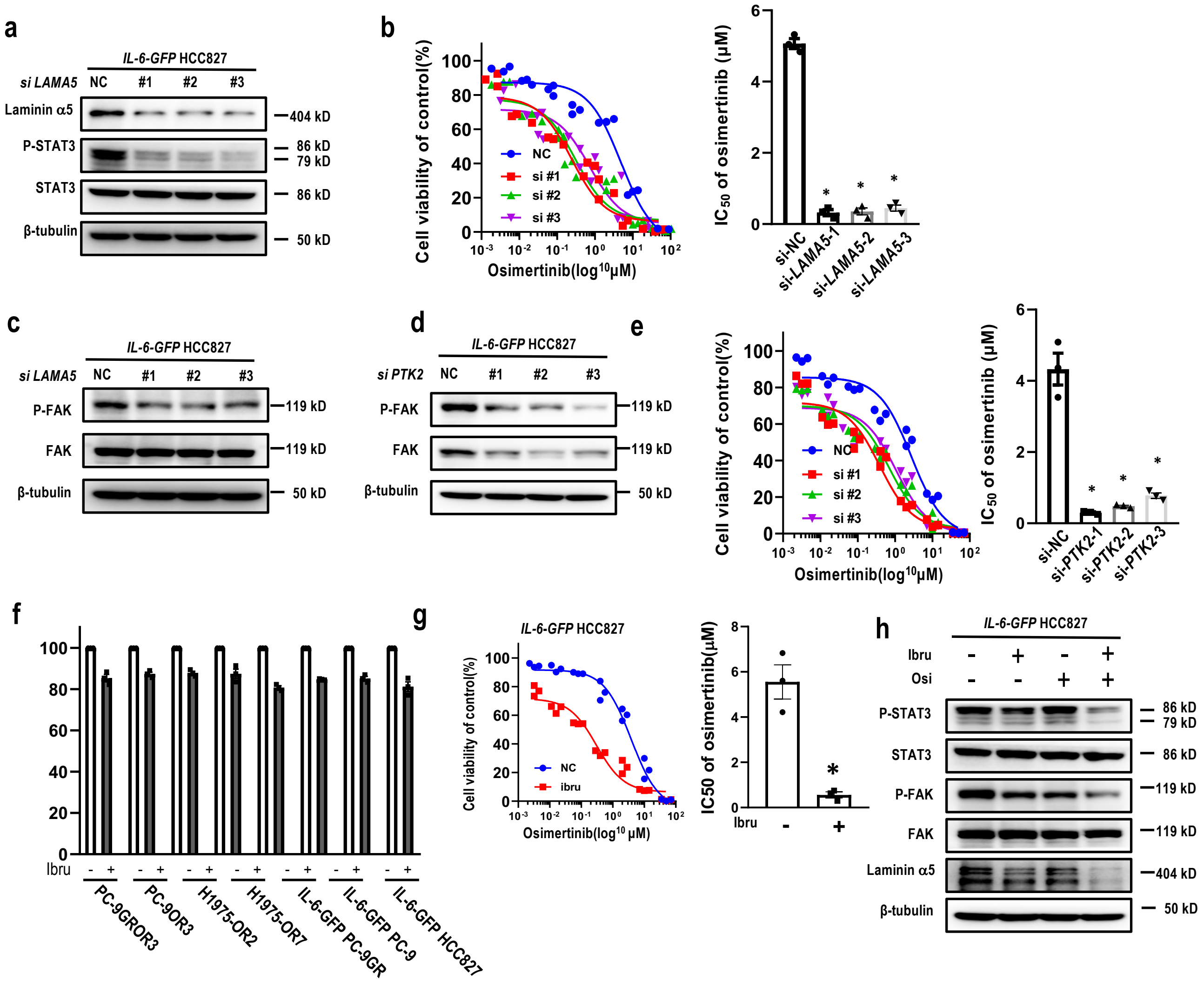

Supplementary Figure 6. Ibrutinib overcame IL-6-induced osimertinib resistance through inhibition Laminin α5/FAK pathway in *IL-6-GFP* HCC827 cells. (a), The levels of Laminin α5 and STAT3 in *IL-6-GFP* HCC827 cells after transfection with *LAMA5* siRNAs, respectively. (b), Cell viability CCK-8 assay for *IL-6-GFP* HCC827 cells transfected with control or *LAMA5* siRNAs, respectively, and treated with increasing concentrations of osimertinib for 48h (n=3 biologically independent experiments). Data are shown as mean ± SEM. Histogram shows IC_{50} values in the indicated groups (*$p < 0.01$ by Student's t-test). (c), Western blot showing the levels of total and phosphorylated PTK2 after transfection with *LAMA5* siRNAs, respectively (d), The levels of total and phosphorylated FAK after transfection with *PTK2* siRNAs, respectively. (e), Cell viability CCK-8 assay for *IL-6-GFP* HCC827 cells transfected with control or *PTK2* siRNAs, respectively, and treated with increasing concentrations of osimertinib for 48h. (f), Cell viability detection of multiple cell lines treated with ibrutinib (0.5μM) for 48h. (g), Cell viability CCK-8 assay for *IL-6-GFP* HCC827 cells treated with ibrutinib (0.5μM) and increasing concentrations of osimertinib for 48h (n=3 biologically independent experiments). Data are expressed as mean ± SEM. (h), Western blot showing the expression levels of indicated proteins in *IL-6-GFP* HCC827 cells in the presence of ibrutinib, osimertinib, or both. β-tubulin served as loading control.

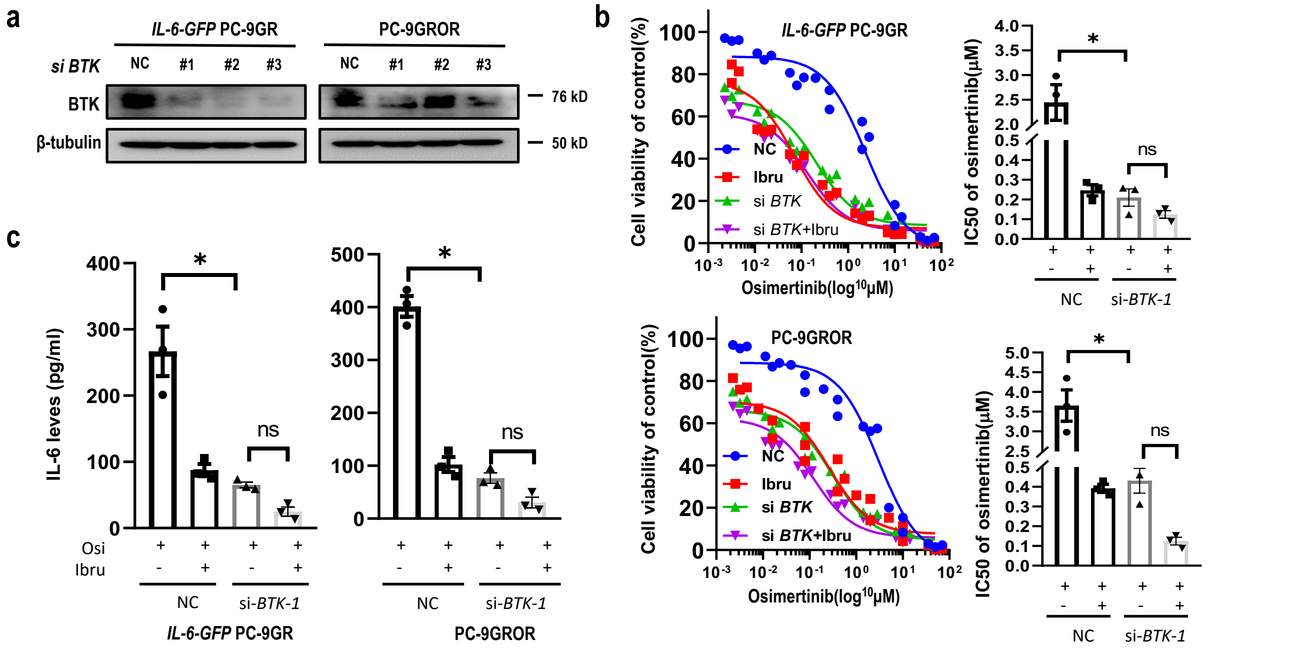

Supplementary Figure 7. Knockdown of *BTK* suppressed the effect of ibrutinib on osimertiinb sensitivity. (a), The levels of BTK in *IL-6-GFP* PC-9GR and PC-9GROR cells after transfection with *BTK* siRNAs, respectively. (b), Cell viability CCK-8 assay for indicated cells transfected with control or BTK siRNAs, respectively, and treated with increasing concentrations of osimertinib for 48h (n=3 biologically independent experiments). Data are shown as mean ± SEM (*p < 0.01 by Student's t-test). (c), ELISA analysis of IL-6 levels in *IL-6-GFP* PC-9GR and PC-9GROR cells transfected with control siRNAs or *BTK* siRNA and treated as indicated (n=3 biologically independent experiments).

Source data for Figure 2E

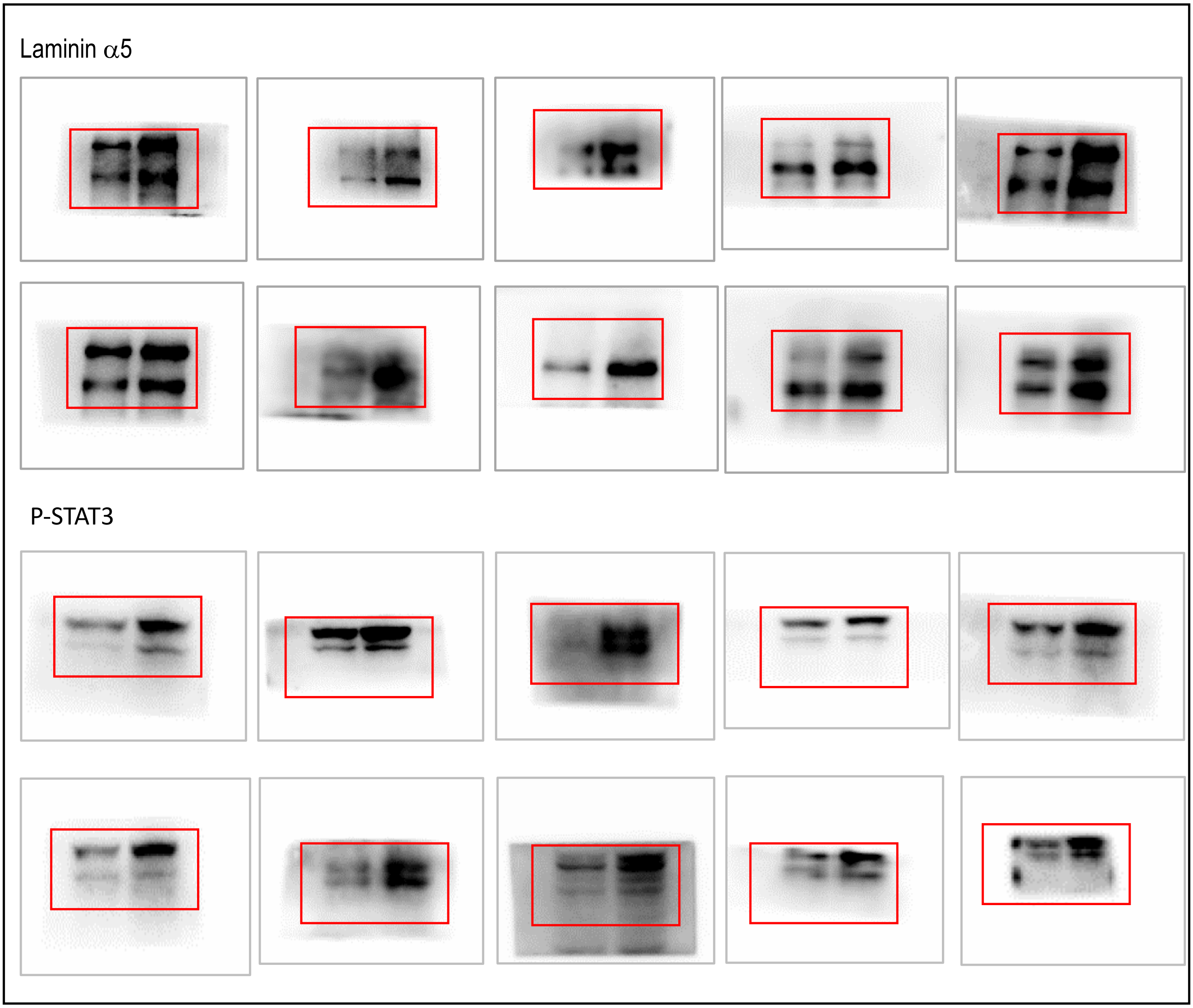

Supplementary Figure 8. Full gel images related to the indicated figures (continued on the next page).

Source data for Figure 2E

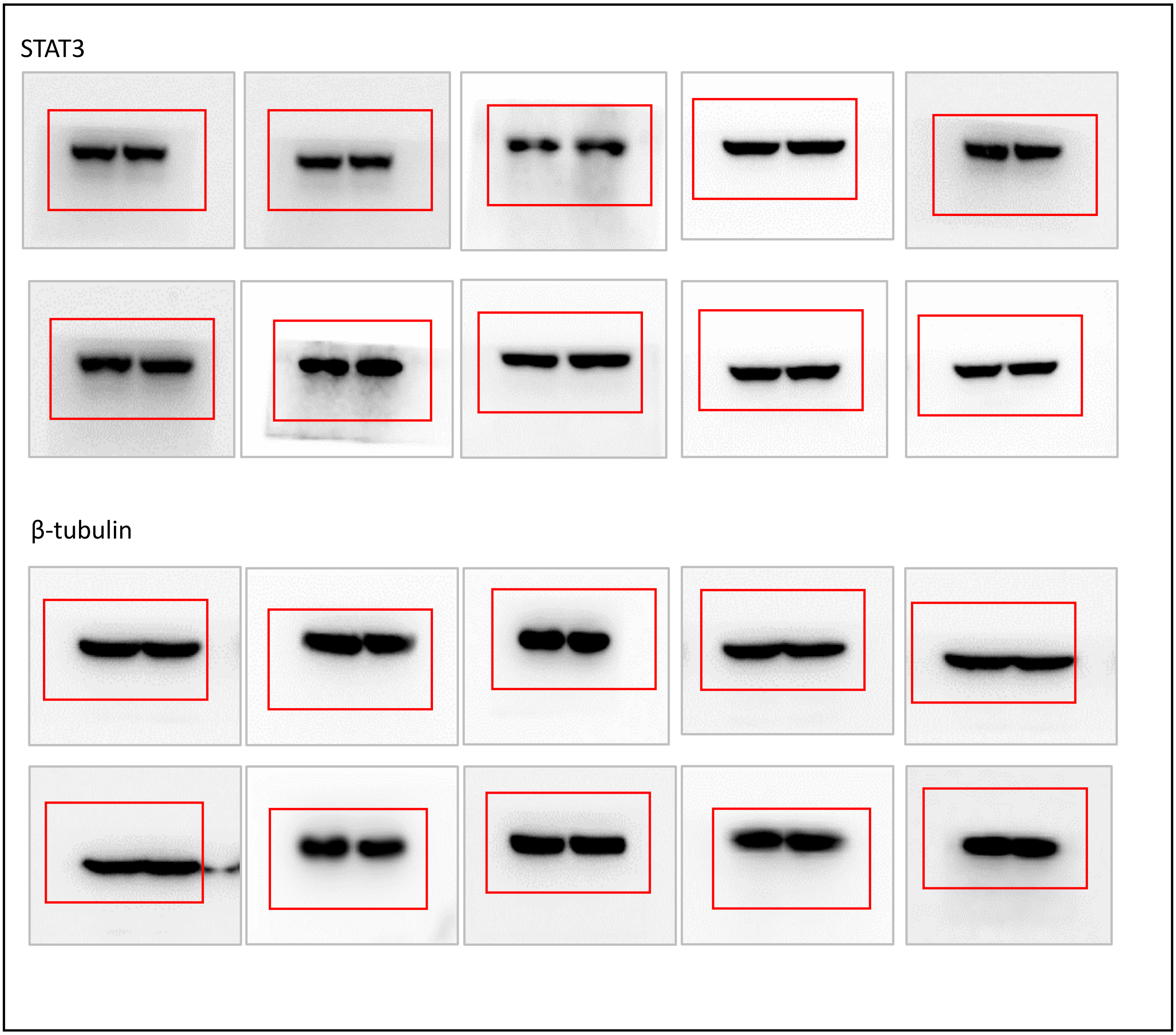

Supplementary Figure 8. Full gel images related to the indicated figures (continued on the next page).

Source data for Figure 2F

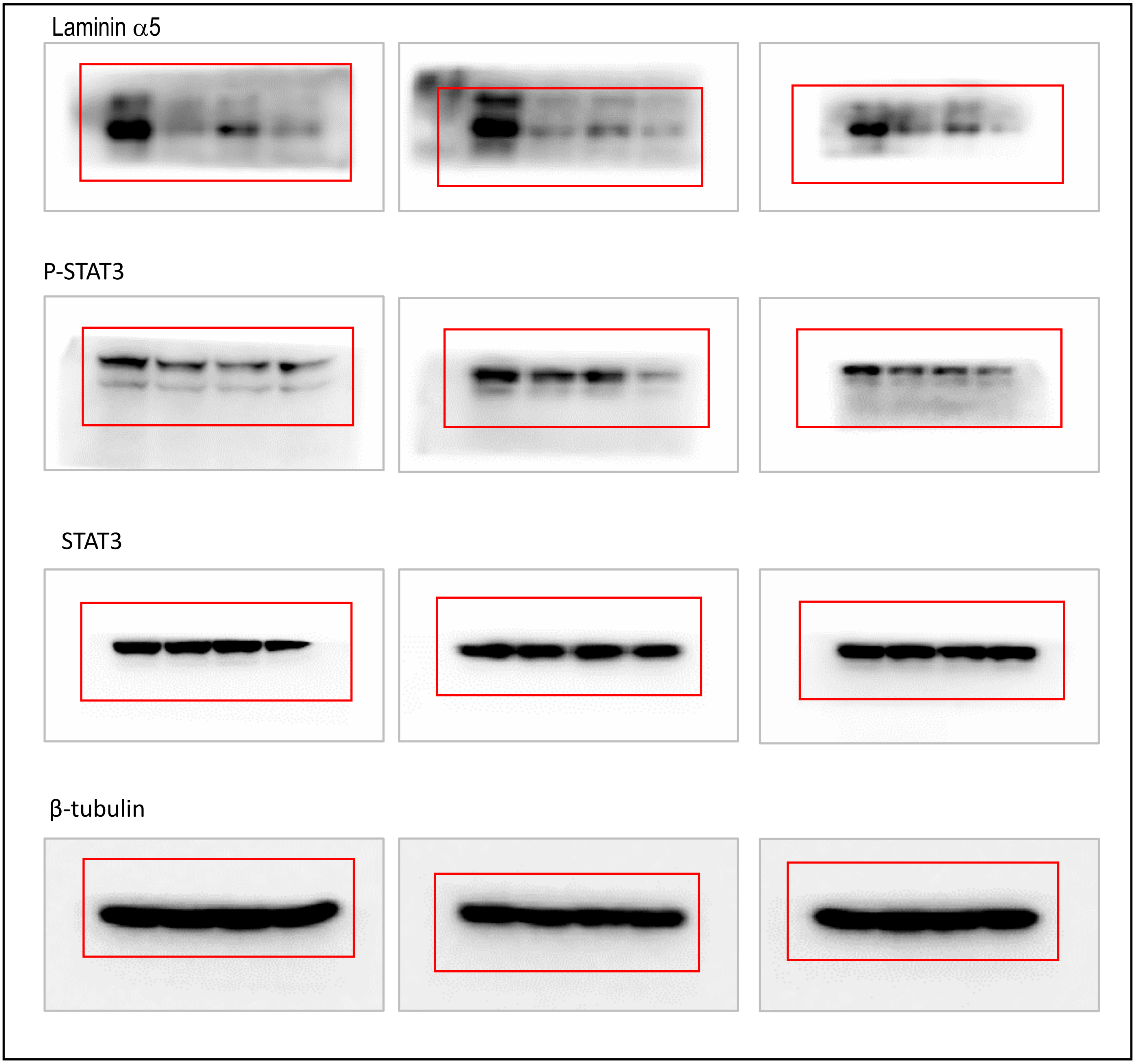

Supplementary Figure 8. Full gel images related to the indicated figures (continued on the next page).

Source data for Figure 3C

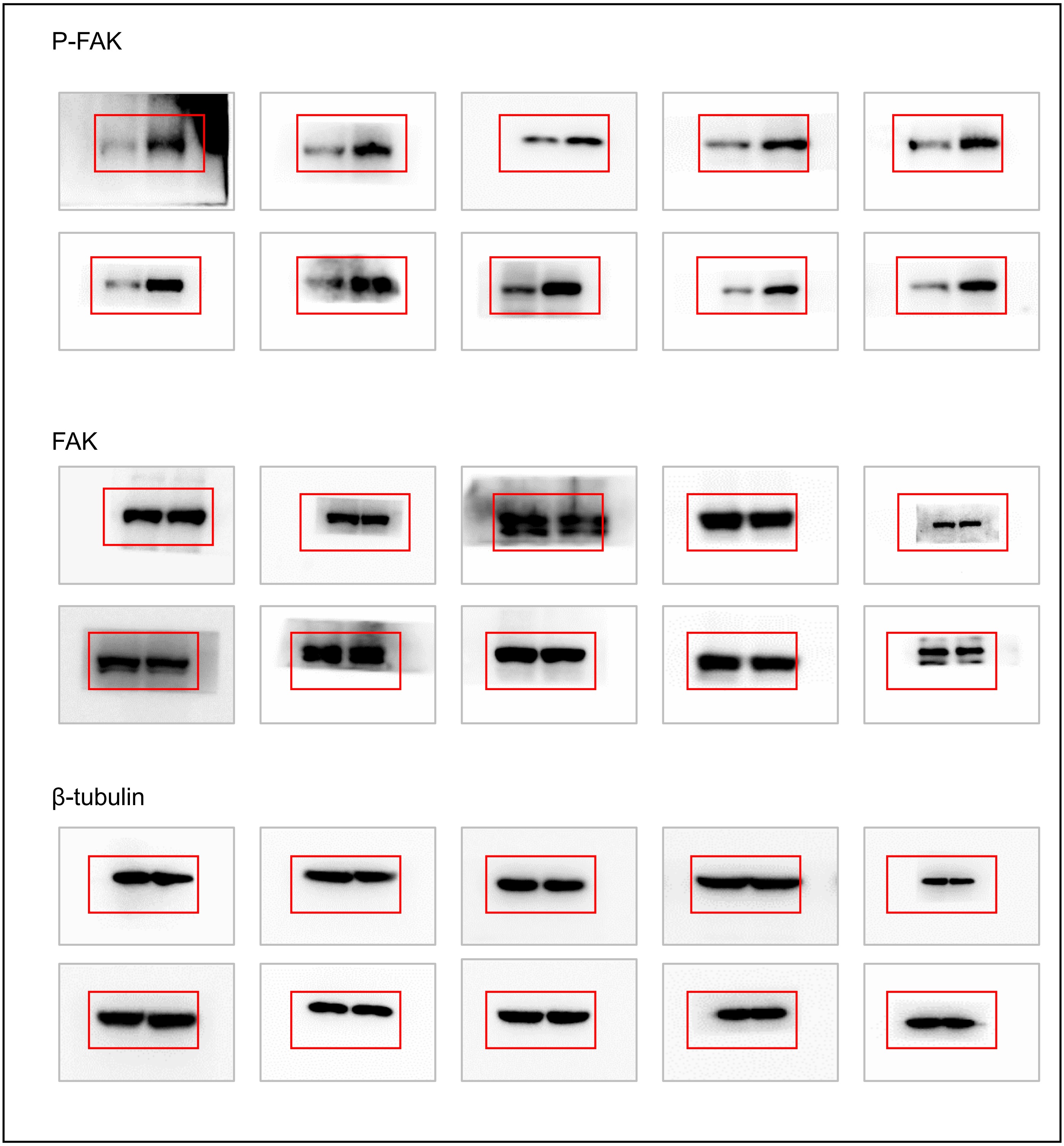

Supplementary Fig. 8. Full gel image related to the indicated figures. (continued on next page)

Source data for Figure 3D

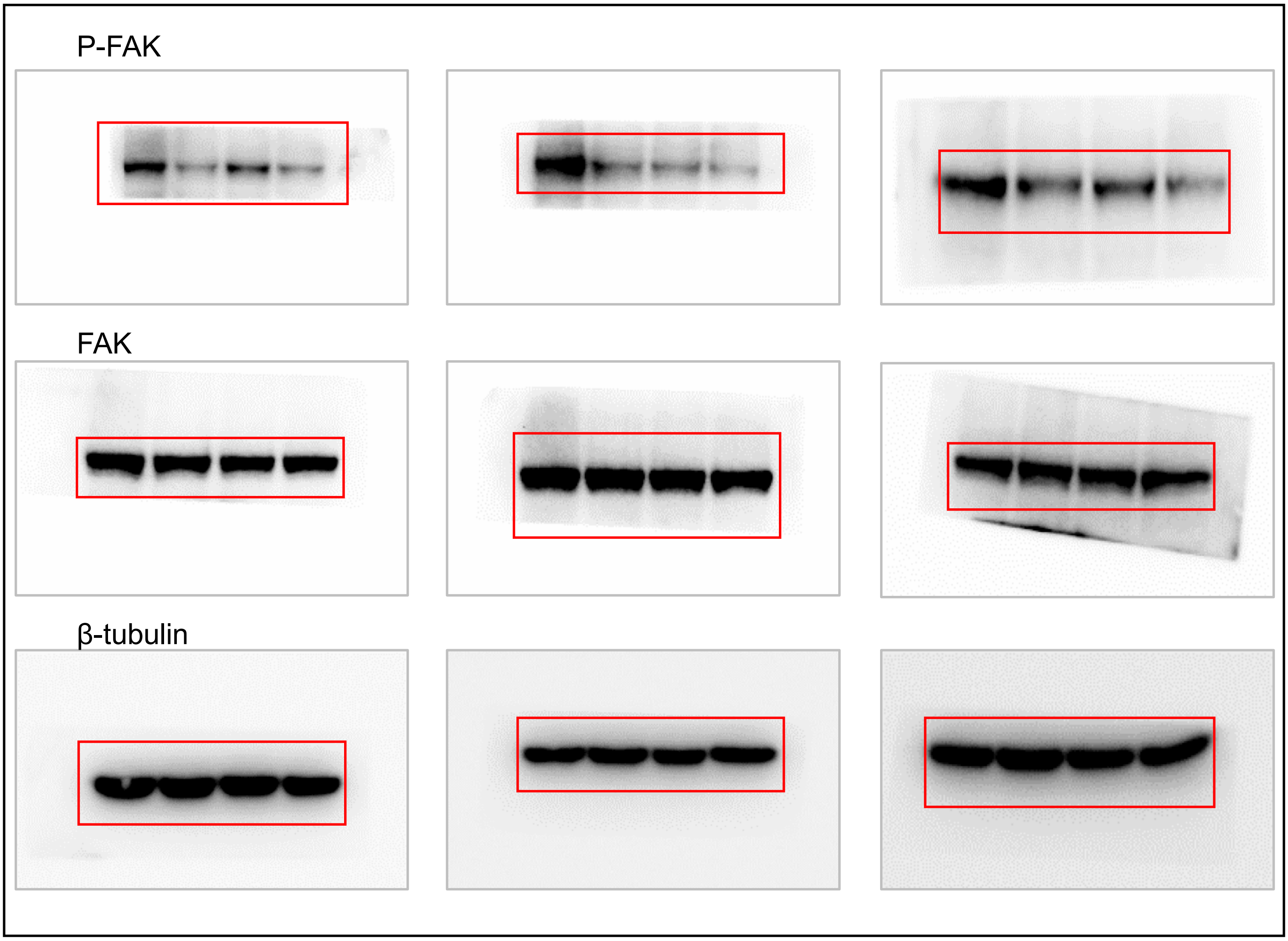

Supplementary Fig. 8. Full gel image related to the indicated figures. **(continued on next page)**

Source data for Figure 3E

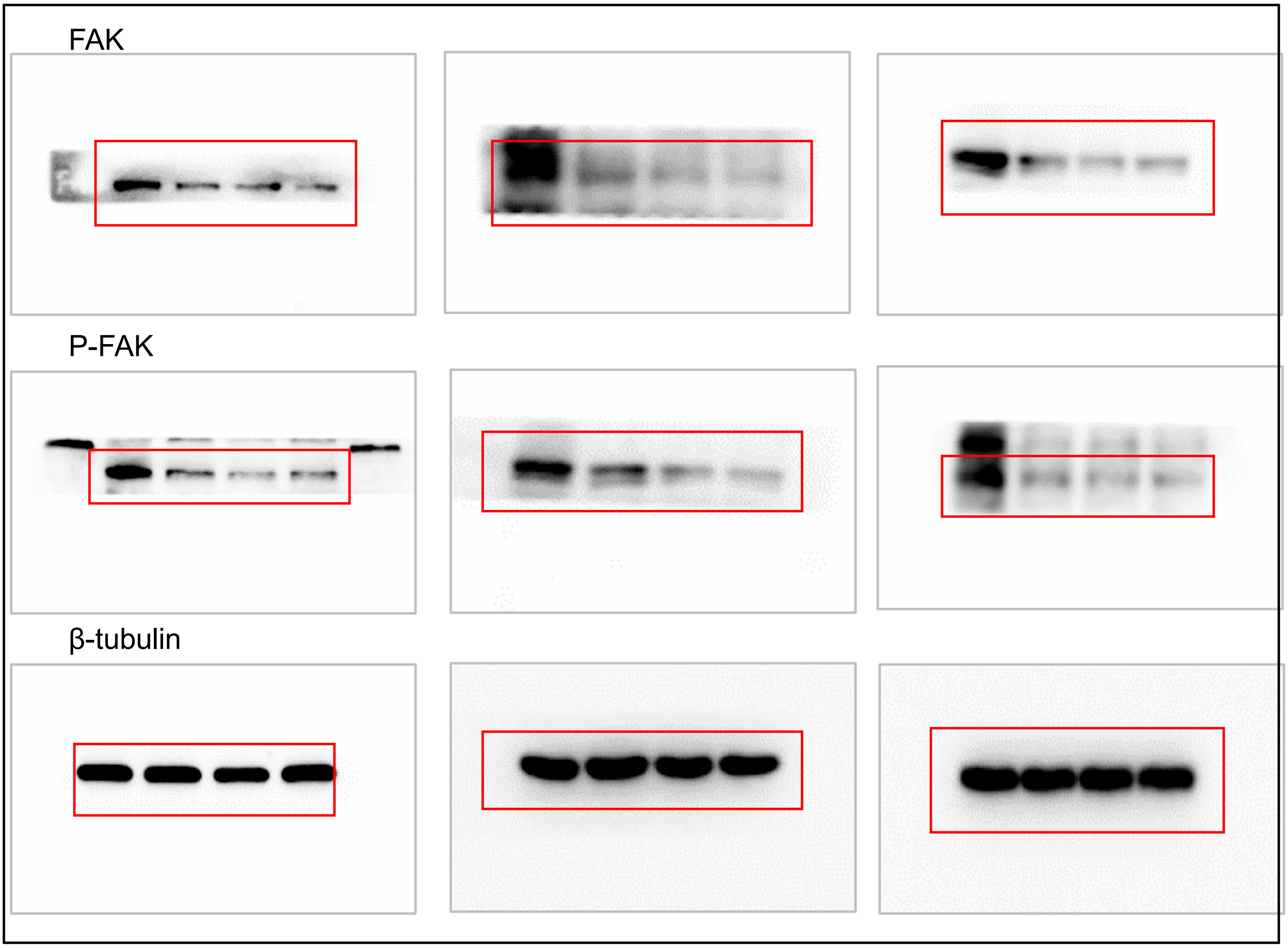

Supplementary Fig. 8. Full gel image related to the indicated figures. **(continued on next page)**

Source data for Figure 4D

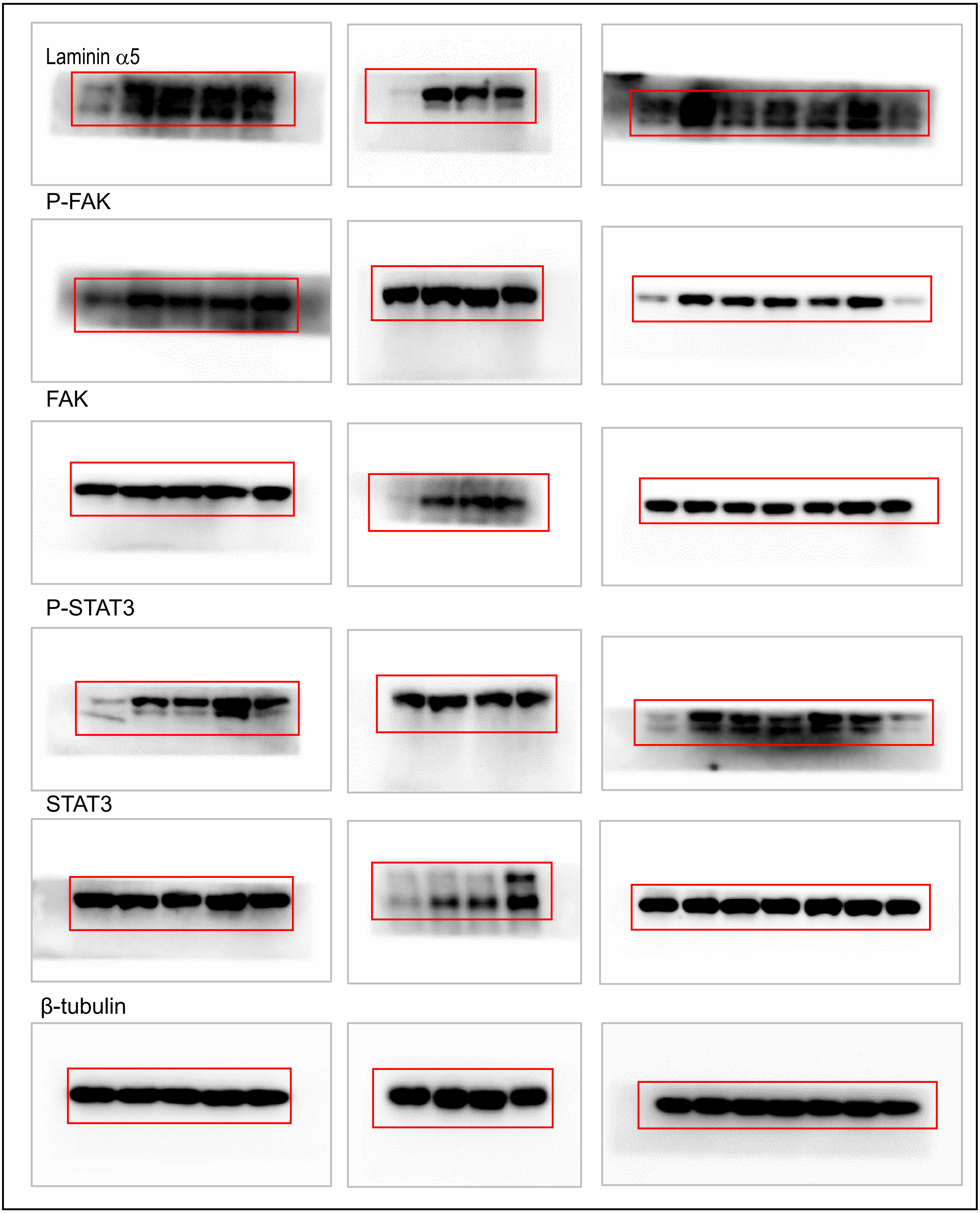

Supplementary Fig. 8. Full gel image related to the indicated figures. **(continued on next page)**

Source data for Figure 4F

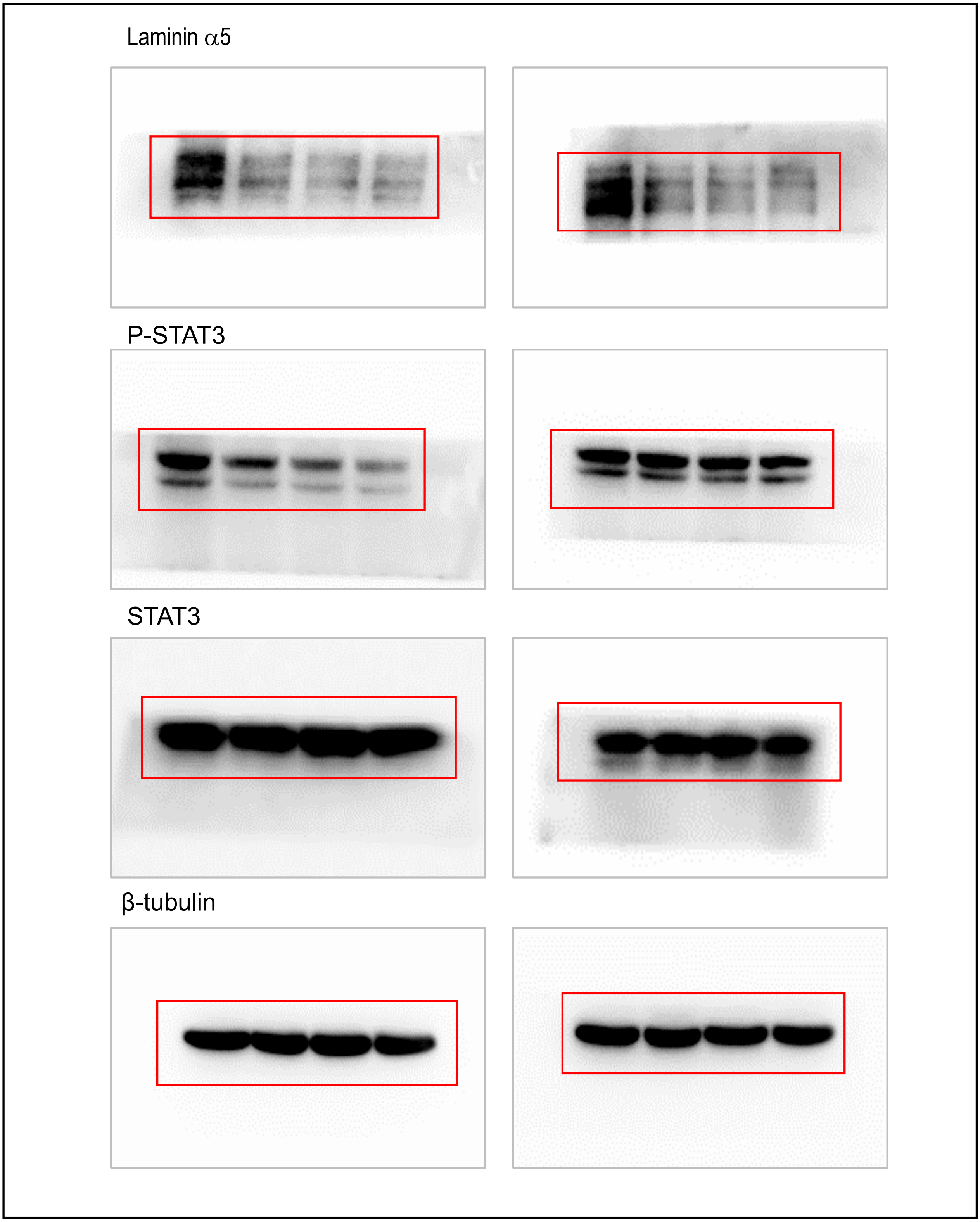

Supplementary Fig. 8. Full gel image related to the indicated figures. (continued on next page)

Source data for Figure 4H

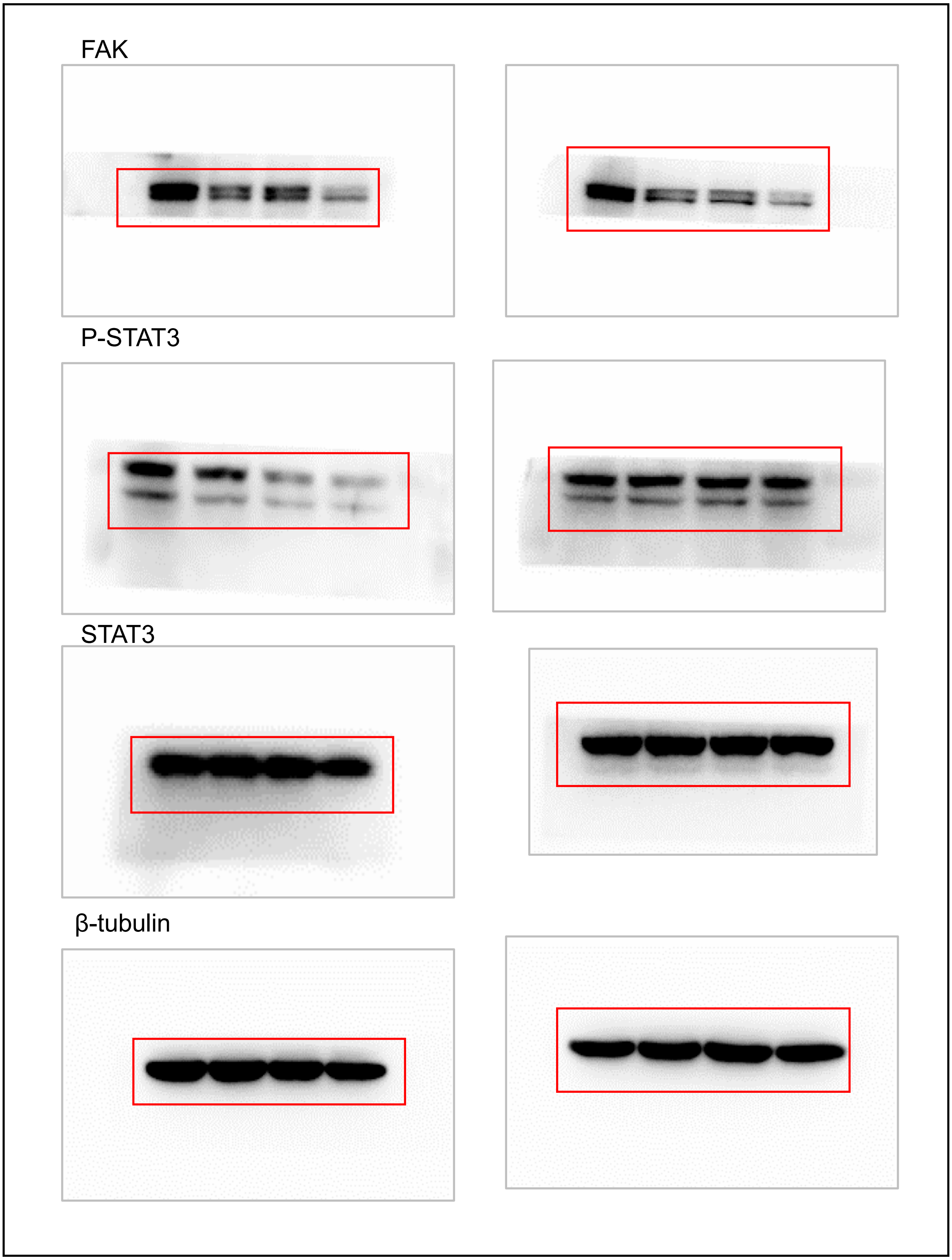

Supplementary Fig. 8. Full gel image related to the indicated figures. (continued on next page)

Source data for Figure 5E

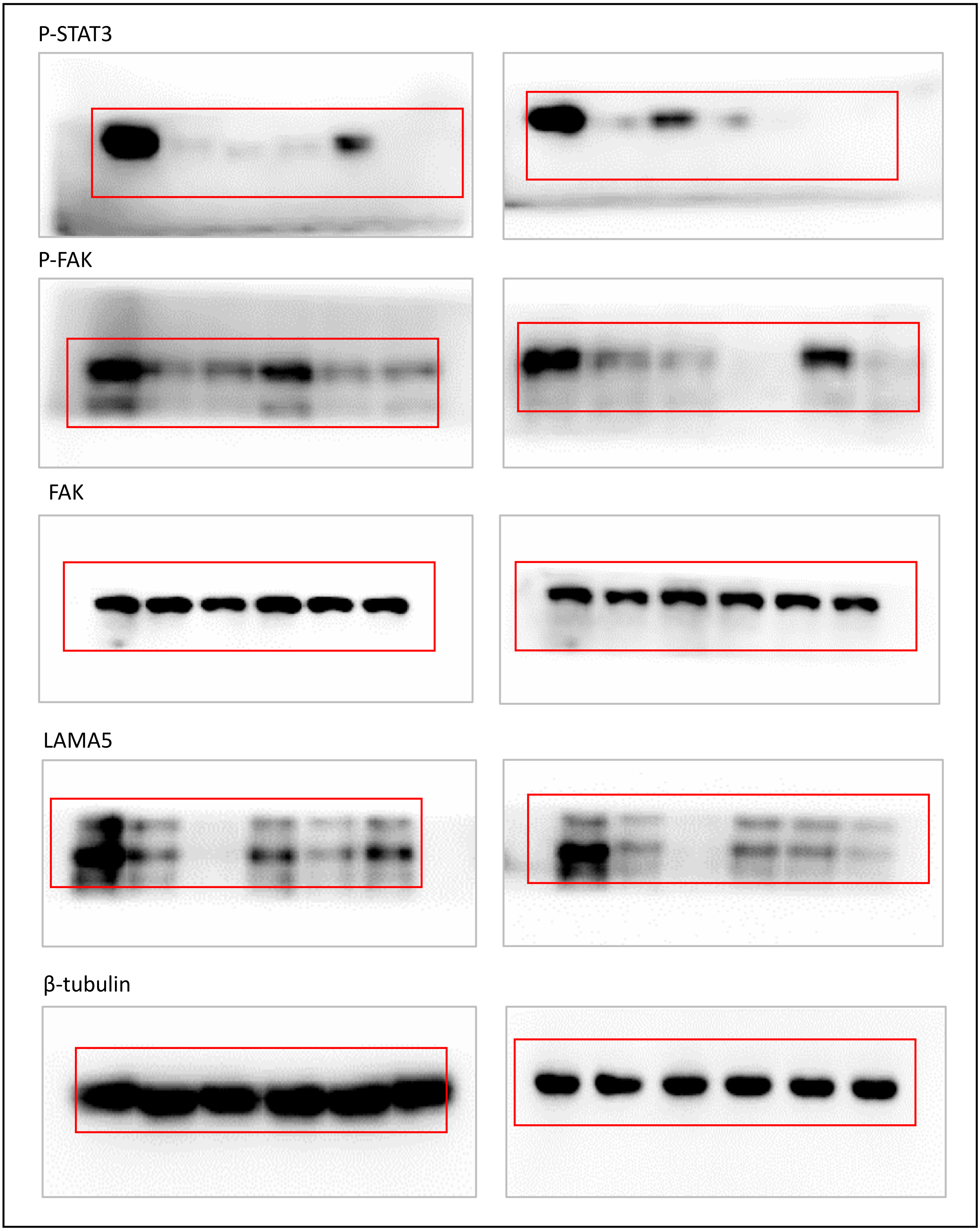

Supplementary Figure 8. Full gel images related to the indicated figures (continued on the next page).

Source data for Figure 6B

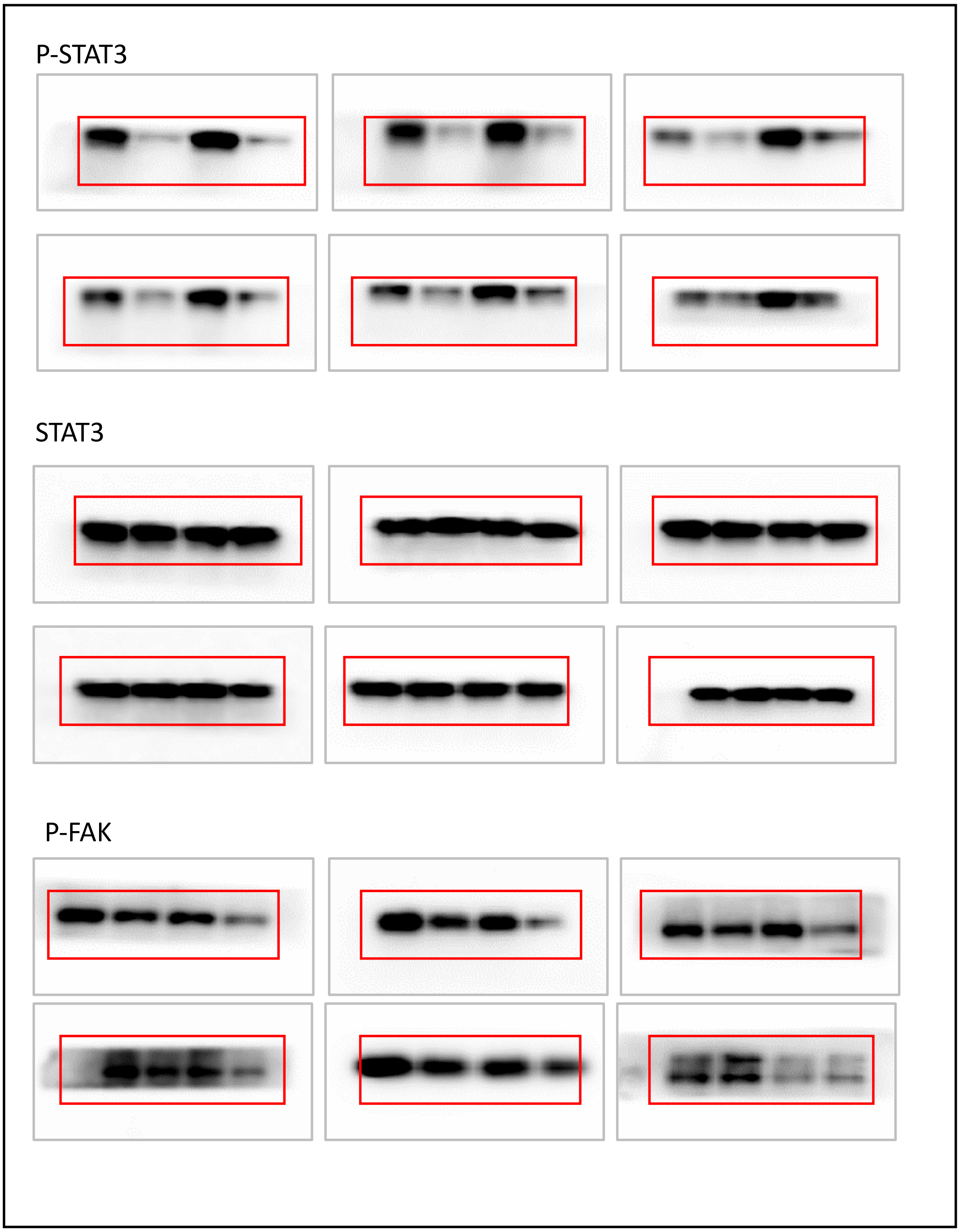

Supplementary Figure 8. Full gel images related to the indicated figures (continued on the next page).

Source data for Figure 6B

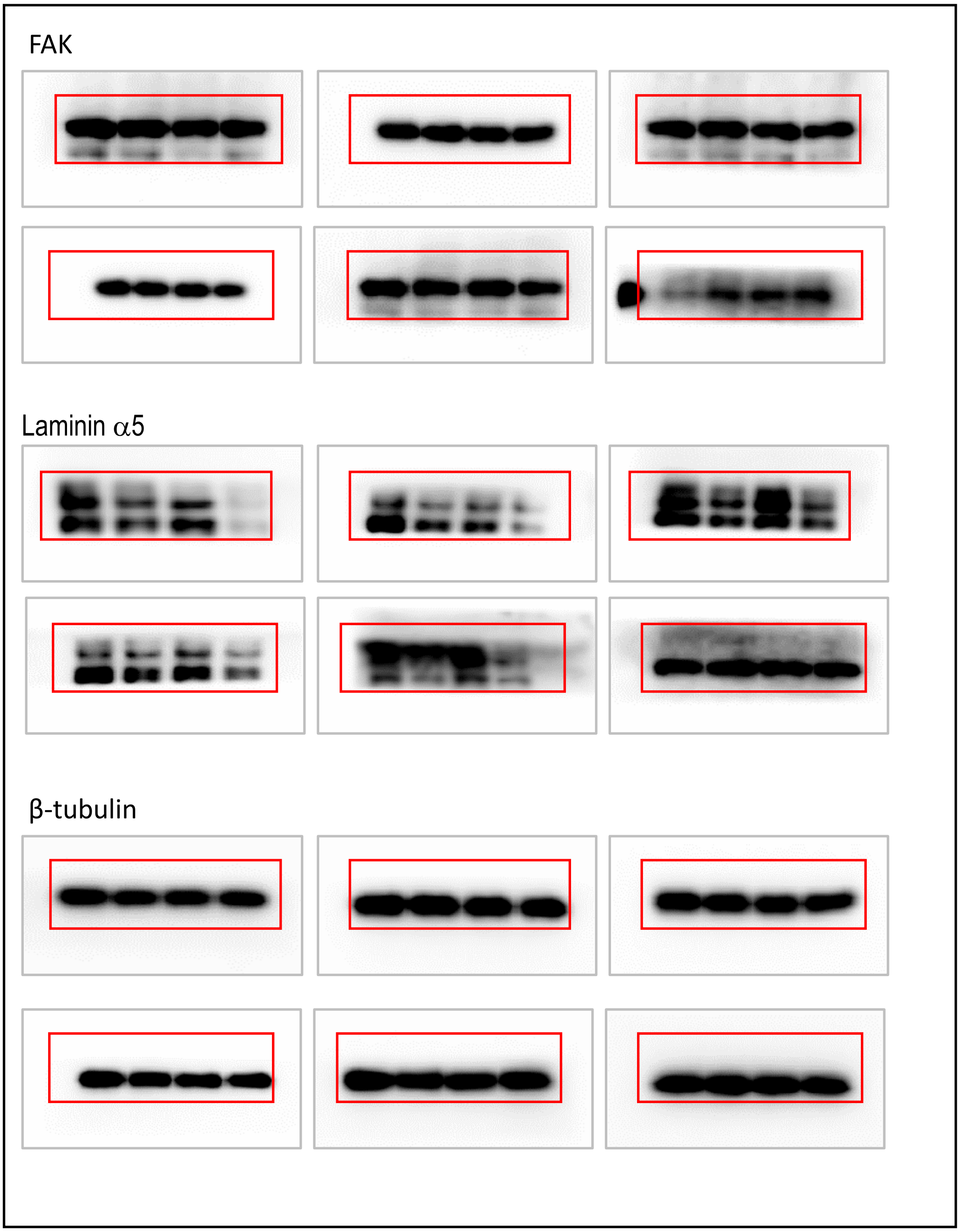

Supplementary Figure 8. Full gel images related to the indicated figures (continued on the next page).

Source data for Supplementary Figure 4A

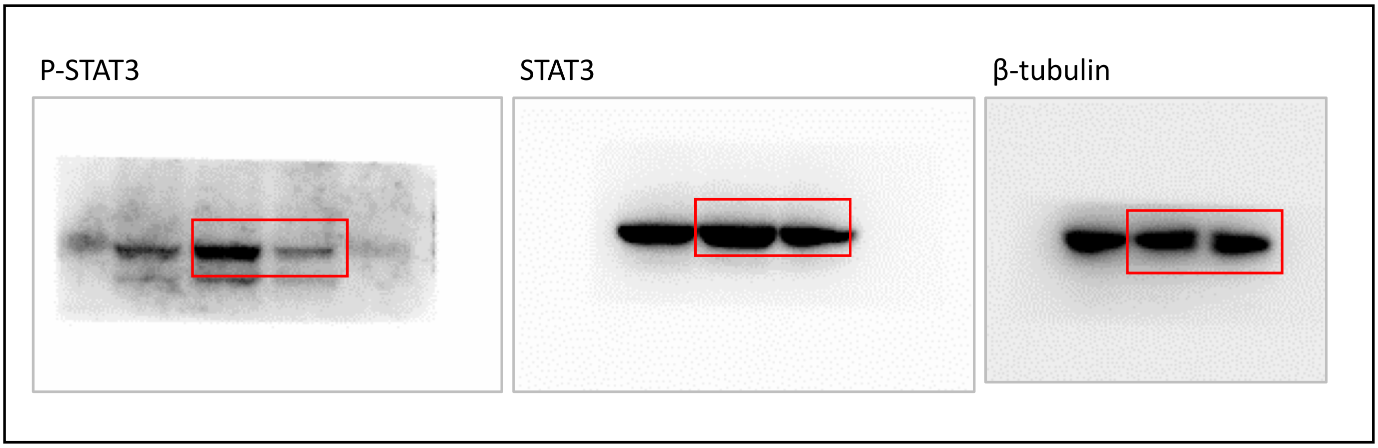

Source data for Supplementary Figure 4C

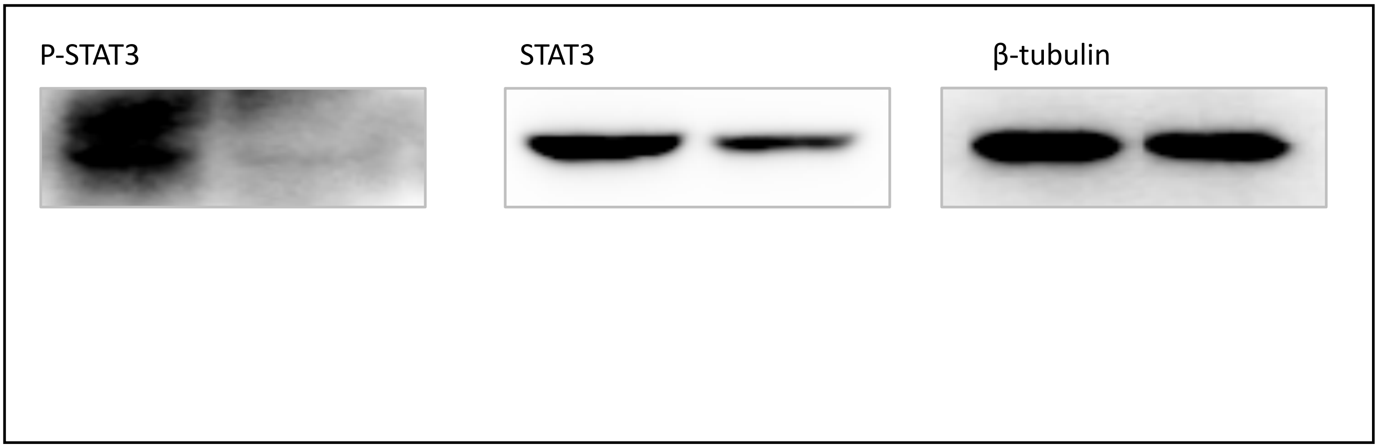

Supplementary Figure 8. Full gel images related to the indicated figures (continued on the next page).

Source data for Supplementary Figure 6A

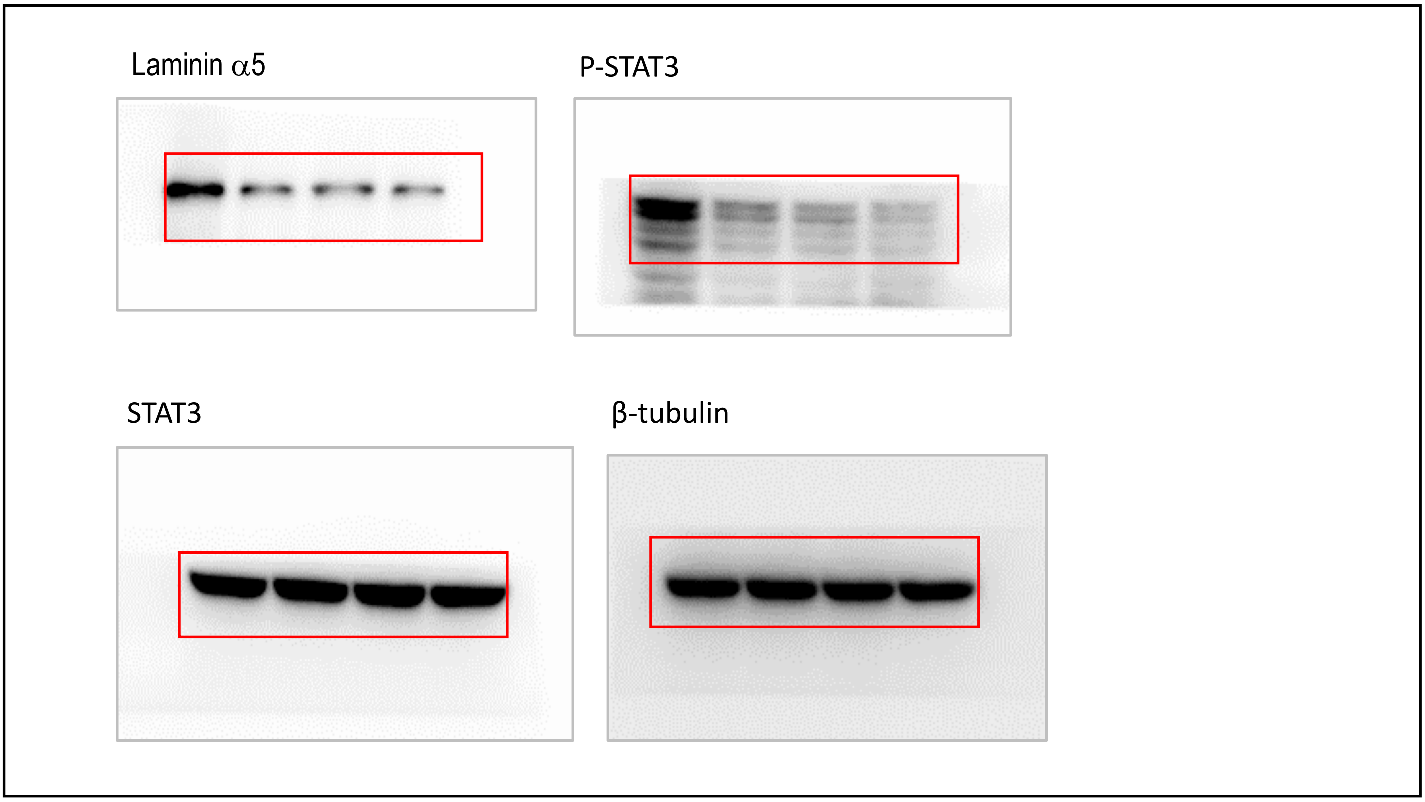

Source data for Supplementary Figure 6C

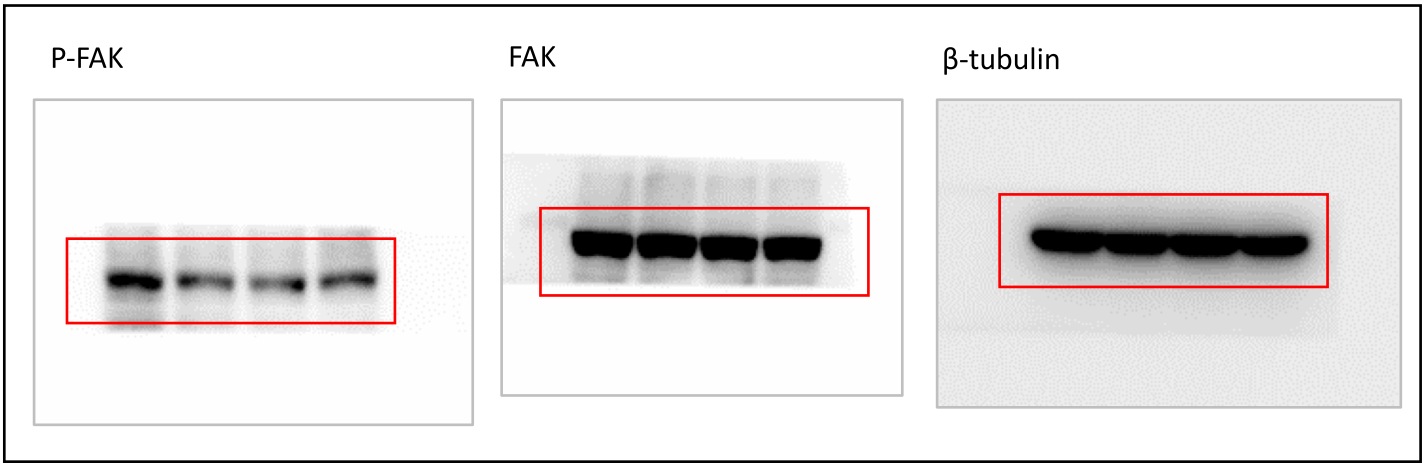

Supplementary Figure 8. Full gel images related to the indicated figures (continued on the next page).

Source data for Supplementary Figure 6D

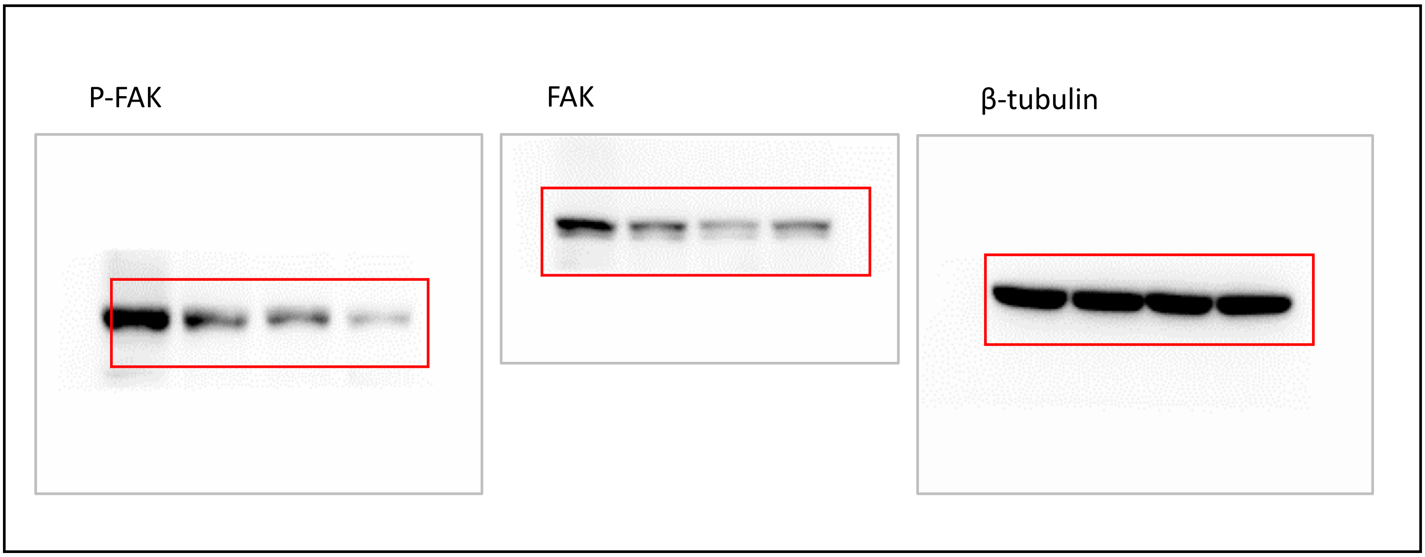

Source data for Supplementary Figure 6H

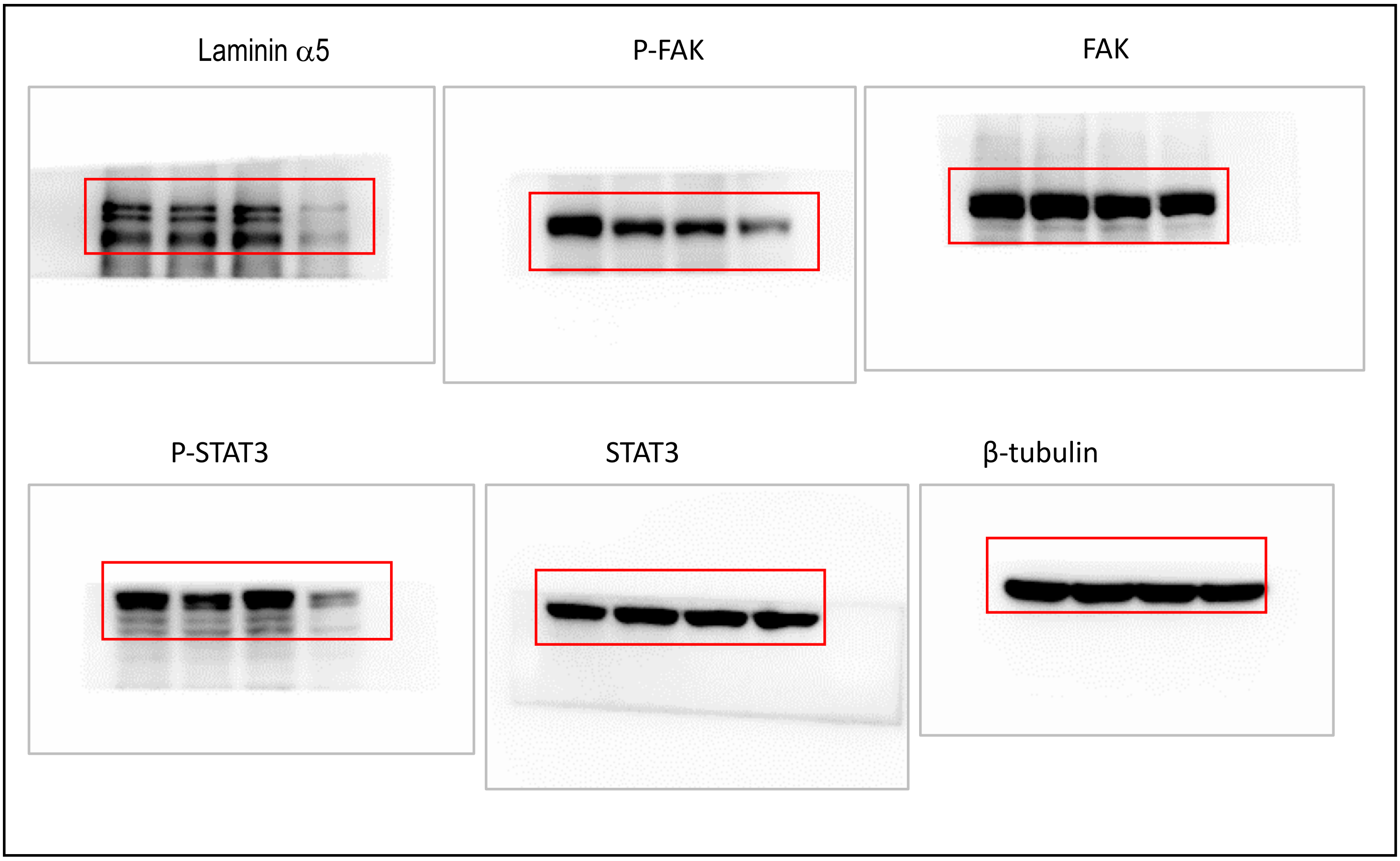

Supplementary Figure 8. Full gel images related to the indicated figures (continued on the next page).

Source data for Supplementary Figure 7A

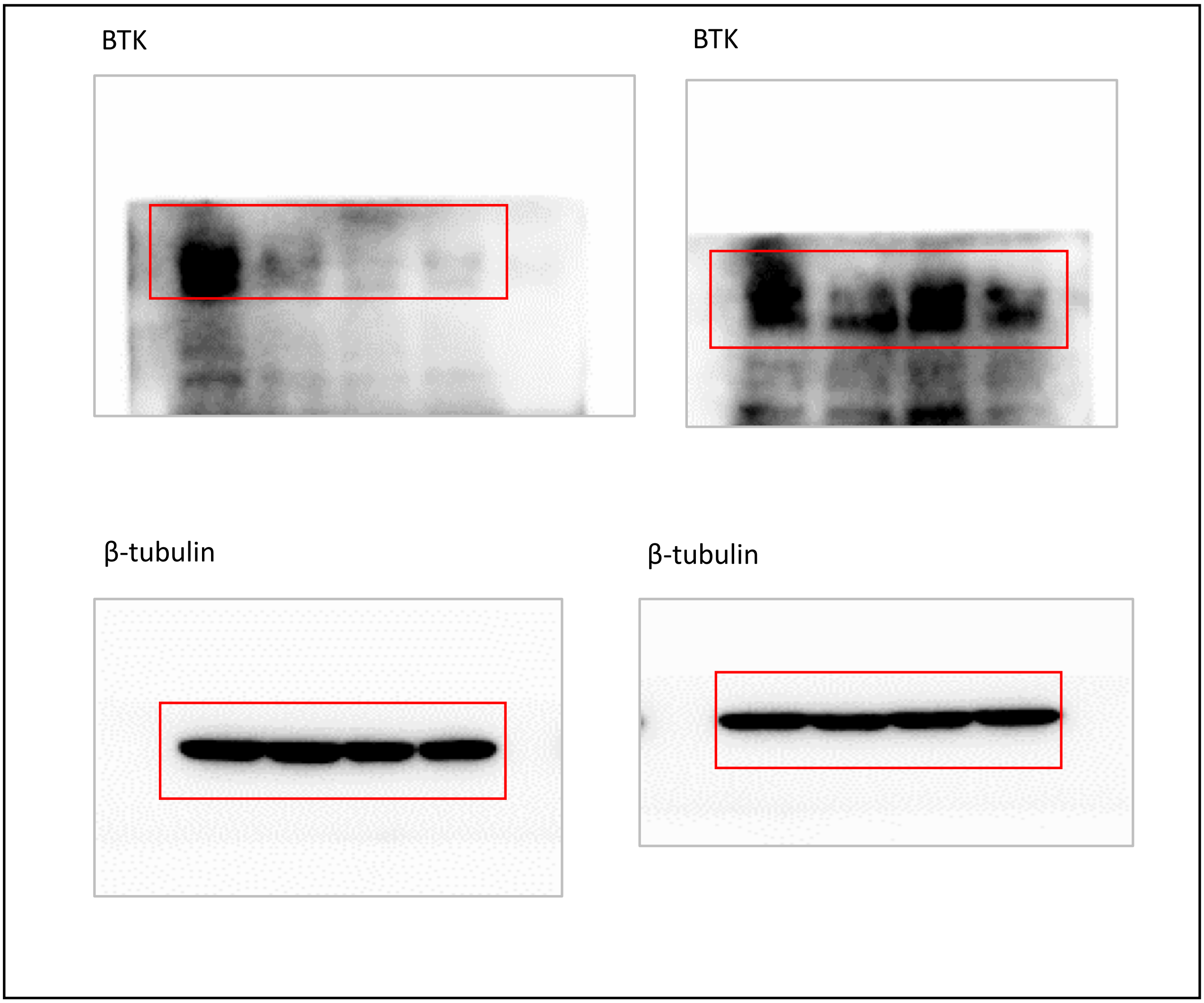

Supplementary Figure 8. Full gel images related to the indicated figures.

Supplementary Table 1 Patient Characterists.

	Total (N=253)	1st line Gefitinib (N=226)	1st line Osimertinib (N=27)
Age - (Mean±SD), years	59.94±11.05	60.08±11.28	58.81±9.01
Sex – No. (%)			
Male	104 (41.11%)	97 (42.92%)	7 (25.93%)
Female	149 (58.89%)	129 (57.08%)	20 (74.07%)
Pathology – No. (%)			
Adenocarcinoma	242 (95.65%)	215 (95.13%)	27 (100%)
Squamous carcinoma	9 (3.56%)	9 (3.98%)	0 (0%)
Adenosquamous carcinoma	2 (0.79%)	2 (0.89%)	0 (0%)
Stage – No. (%)			
III	35 (13.83%)	33 (14.60%)	3 (11.11%)
IV	218 (86.17%)	193 (85.40%)	24 (88.89%)
Metastasis sites – No. (%)			
Lung	99 (39.13%)	91 (40.27%)	8 (29.63%)
Lymph nodes	131 (51.78%)	121 (53.54%)	10 (37.04%)
Pleura	104 (41.11%)	94 (41.59%)	10 (37.04%)
Bone	139 (54.94%)	125 (55.31%)	14 (51.85%)
Brain	48 (18.97%)	34 (15.04%)	14 (51.85%)
Liver	35 (13.83%)	31 (13.72%)	4 (14.81%)
Abdominal	5 (1.98%)	4 (1.77%)	1 (3.70%)
Pericardium	18 (7.11%)	15 (6.64%)	3 (11.11%)
kidney	7 (2.77%)	7 (3.10%)	0 (0%)
Spleen	4 (1.58%)	4 (1.77%)	0 (0%)
Other sites	30 (11.86%)	27 (11.95%)	3 (11.11%)
Types of EGFR mutations – No. (%)			
G719X	7 (2.77%)	6 (2.65%)	1 (3.70%)
19DEL	134 (52.96%)	120 (53.10%)	14 (51.85%)
S768I	1 (0.40%)	1 (0.44%)	0 (0%)
T790M	8 (3.16%)	0 (0%)	8 (29.63%)
C797S	0 (0%)	0 (0%)	0 (0%)
L858R	109 (43.08%)	101 (44.69%)	8 (29.63%)
L861Q	4 (1.58%)	3 (1.33%)	1 (3.70%)

Supplementary Table 2: List of primary and secondary antibodies used in the study.

Antibody	Species raised	IHC dilution	WB dilution	IF dilution	Product Code	Source
P-FAK	Rabbit polyclonal	1:50	1:1000	1:100	ab81298	https://www.abcam.cn/fak-phospho-y397-antibody-ep2160y-ab81298.html
FAK	Rabbit polyclonal		1:1000		ab40794	https://www.abcam.cn/fak-antibody-ep695y-ab40794.html
P-STAT3	Rabbit polyclonal		1:1000		#9145	https://www.cellsignal.cn/products/primary-antibodies/phospho-stat3-tyr705-d3a7-xp-rabbit-mab/9145?site-search-type=Products&N=4294956287&Ntt=%239145&fromPage=plp&_requestid=4742194
STAT3	Rabbit polyclonal		1:1000		#12640	https://www.cellsignal.cn/products/primary-antibodies/stat3-d3z2g-rabbit-mab/12640?site-search-type=Products&N=4294956287&Ntt=%2312640&fromPage=plp&_requestid=4742233
β-tubulin	Rabbit polyclonal		1:1000		#A5032	http://www.bimake.cn/antibody/beta-tubulin-rabbit-recombinant-mab.html
Laminin α5	Mouse polyclonal			1:100	#ab77175	https://www.abcam.cn/products?keywords=%23ab77175
Laminin α5	Rabbit polyclonal	1:50	1:1000		#ab184330	https://www.abcam.cn/products?keywords=%23ab184330
IL-6	Rabbit polyclonal	1:50			TA500067	http://www.zsbio.com/product/TA500067
Goat AntiRabbit Immunoglobulins/HRP	Goat polyclonal		1:10000		SSA005	https://cn.sinobiological.com/antibodies/secondary-antibody-goat-anti-rat-igg-hrp-ssa005
Goat Anti-rabbit IgG H&L/FITC	Goat polyclonal			1:100	bs-0295G-FITC	http://www.bioss.com.cn/prolook_03.asp?id=AF08169606011201&pro37=4
Rabbit Anti-Mouse IgG H&L/Cy3	Goat polyclonal			1:100	bs-0296R-Cy3	http://www.bioss.com.cn/prolook_03.asp?id=AF08169606011809&pro37=4
