# Supplementary material for: Ibrutinib reverses IL-6-induced osimertinib resistance through inhibition of Laminin α5/FAK signaling
Source: Commun Biol. 2022 Feb 23;5:155. doi: 10.1038/s42003-022-03111-7 (PMC8866396; doi:10.1038/s42003-022-03111-7)
Supplement: Supplementary file 3 — Description of Additional Supplementary Files [file 42003_2022_3111_MOESM3_ESM.pdf]

Description of Additional Supplementary Files

File name: Supplementary Data 1
Description: Raw data of RNA sequencing on paired osimertinib sensitive and resistant cells.

File name: Supplementary Data 2
Description: Raw data of proteomics on paired osimertinib sensitive and resistant cells.

File name: Supplementary Data 3
Description: Raw data of small molecular sequencing.

File name: Supplementary Data 4
Description: Source Data for Figures and Supplementary Figures.
