# Supplementary material for: Ibrutinib reverses IL-6-induced osimertinib resistance through inhibition of Laminin α5/FAK signaling
Source: Commun Biol. 2022 Feb 23;5:155. doi: 10.1038/s42003-022-03111-7 (PMC8866396; doi:10.1038/s42003-022-03111-7)
Supplement: Supplementary file 8 — Reporting Summary [file 42003_2022_3111_MOESM8_ESM.pdf]

nature research

Corresponding author(s): Yong He, Chen Hu, Li Li

Last updated by author(s): Dec 26, 2021

Reporting Summary

Nature Research wishes to improve the reproducibility of the work that we publish. This form provides structure for consistency and transparency in reporting. For further information on Nature Research policies, see our Editorial Policies and the Editorial Policy Checklist.

Statistics

For all statistical analyses, confirm that the following items are present in the figure legend, table legend, main text, or Methods section.

n/a	Confirmed	
☐	☒	The exact sample size (*n*) for each experimental group/condition, given as a discrete number and unit of measurement
☐	☒	A statement on whether measurements were taken from distinct samples or whether the same sample was measured repeatedly
☐	☒	The statistical test(s) used AND whether they are one- or two-sided<br>*Only common tests should be described solely by name; describe more complex techniques in the Methods section.*
☒	☐	A description of all covariates tested
☐	☒	A description of any assumptions or corrections, such as tests of normality and adjustment for multiple comparisons
☐	☒	A full description of the statistical parameters including central tendency (e.g. means) or other basic estimates (e.g. regression coefficient) AND variation (e.g. standard deviation) or associated estimates of uncertainty (e.g. confidence intervals)
☐	☒	For null hypothesis testing, the test statistic (e.g. *F*, *t*, *r*) with confidence intervals, effect sizes, degrees of freedom and *P* value noted<br>*Give P values as exact values whenever suitable.*
☒	☐	For Bayesian analysis, information on the choice of priors and Markov chain Monte Carlo settings
☒	☐	For hierarchical and complex designs, identification of the appropriate level for tests and full reporting of outcomes
☒	☐	Estimates of effect sizes (e.g. Cohen's *d*, Pearson's *r*), indicating how they were calculated

Our web collection on statistics for biologists contains articles on many of the points above.

Software and code

Policy information about availability of computer code

Data collection	CytExpert version 2.3<br>Skanlt Software 5.0
Data analysis	Graphpad Prism version 8.0

For manuscripts utilizing custom algorithms or software that are central to the research but not yet described in published literature, software must be made available to editors and reviewers. We strongly encourage code deposition in a community repository (e.g. GitHub). See the Nature Research guidelines for submitting code & software for further information.

Data

Policy information about availability of data

All manuscripts must include a data availability statement. This statement should provide the following information, where applicable:

- Accession codes, unique identifiers, or web links for publicly available datasets
- A list of figures that have associated raw data
- A description of any restrictions on data availability

All data supporting the findings of this study are available within the article and its Supplementary Information files and from the corresponding authors upon reasonable request.

Field-specific reporting

Please select the one below that is the best fit for your research. If you are not sure, read the appropriate sections before making your selection.

☒ Life sciences ☐ Behavioural & social sciences ☐ Ecological, evolutionary & environmental sciences

For a reference copy of the document with all sections, see nature.com/documents/nr-reporting-summary-flat.pdf

Life sciences study design

All studies must disclose on these points even when the disclosure is negative.

Sample size
The sample size of plasma IL-6 levels was not predetermined and all available samples were precessed.

Data exclusions
No data were excluded.

Replication
All data presented were obtained from three or two independent experiments with similar outcomes. (See Figure legends and Methods)

Randomization
For in vitro experiments, cells were seeded identically at the onset of the experiments and randomized into the various treatment groups prior to the beginning of treatment protocols. For mice experiments, we randomized the mice into the each treatment group prior starting treatment.

Blinding
All experiments were not performed blind. Each experiment was designed with proper controls, and samples for comparison were collected and analyzed under the same conditions

Reporting for specific materials, systems and methods

We require information from authors about some types of materials, experimental systems and methods used in many studies. Here, indicate whether each material, system or method listed is relevant to your study. If you are not sure if a list item applies to your research, read the appropriate section before selecting a response.

Materials & experimental systems

n/a	Involved in the study	
☐	☒	Antibodies
☐	☒	Eukaryotic cell lines
☒	☐	Palaeontology and archaeology
☐	☒	Animals and other organisms
☐	☒	Human research participants
☒	☐	Clinical data
☒	☐	Dual use research of concern

Methods

n/a	Involved in the study	
☒	☐	ChIP-seq
☐	☒	Flow cytometry
☒	☐	MRI-based neuroimaging

Antibodies

Antibodies used
Primary antibodies for western blot:
anti-STAT3 Ab:Cell Signaling Technology,Cat#12640 , Lot: 28
anti-phospho-STAT3 Ab(Tyr705):Cell Signaling Technology,Cat#9145 , Lot: 24
anti-phospho-FAK Ab(Y397):abcam,Cat# ab81298, Lot:6
anti-FAK Ab:abcam,Cat# ab40794, Lot:16
anti-LAMA5 Ab:abcam,Cat# ab184330, Lot:7
anti-β-tubulin Ab: bimake,Cat# A5032,Lot:18

Secondary antibodies for western blot:
anti-rabbit IgG Ab: Cell Signaling Technology, Cat# 7074, Lot: 23

Primary antibodies for Immunofluorescence:
anti-phospho-FAK Ab(Y397):abcam,Cat# ab81298, Lot:10
anti-LAMA5 Ab:abcam,Cat# ab77175, Lot:15

Secondary antibodies for Immunofluorescence:
anti-Rabbit IgG H&L (Cy3),bioss,Cat# bs-0295P,Lot:2
anti-mouse IgG H&L (Alexa Fluor 488),bioss, Cat#bs-0296G,Lot:5

Validation
All antibodies were obtained commercially and had been validated by the companies

Eukaryotic cell lines

Policy information about cell lines

Cell line source(s)	PC-9 cell line (purchased from ATCC)<br>H1975 cell line (purchased from ATCC)<br>HCC827 cell line (purchased from ATCC)<br>H3255 cell line (purchased from ATCC)<br>PC-9GR cell line (kindly gifted by Prof. J. Xu and Dr. M. Liu from Guangzhou Medical University)<br>PC-9GROR cells were established from PC-9GR<br>PC-9OR cells were established from PC-9<br>H1975-OR cells were established from H1975
Authentication	Cells were obtained from public bioresources bank or company with the information of authentication, and gifted cells were examined by STR analysis before making the cell stock
Mycoplasma contamination	Mycoplasma was not detected in any cell line by the PCR based assay kit.
Commonly misidentified lines (See ICLAC register)	In this study, we did not use any commonly misidentified cell lines .

Animals and other organisms

Policy information about studies involving animals; ARRIVE guidelines recommended for reporting animal research

Laboratory animals	Balb-c/nu, females, 6 weeks of age .
Wild animals	The study did not involve wild animals.
Field-collected samples	The study did not involve samples collected from the field.
Ethics oversight	All mice studies were conducted in line with the protocols approved by the Committee for the Use and Care of experimental animals of the Army Medical University

Note that full information on the approval of the study protocol must also be provided in the manuscript.

Human research participants

Policy information about studies involving human research participants

Population characteristics	Patient characteristics are outlined in Supplementary Table 1.
Recruitment	Patients with histologically-confirmed advanced EGFR-mutant NSCLC who had been treated with EGFR-TKIs at Daping Hospital, Army Medical University, from March 1st 2014 to Dec 31th 2020, were recruited. Those with baseline blood IL-6 levels were included, and those with unknown mutational status, or confirmed bacterial infection, or those without response evaluation were excluded. Serum IL-6 level was retrieved from medical records, which had been measured by Enzyme-linked immunosorbent assay (ELISA) at Department of Clinical Laboratory, Daping Hospital in clinical tests.
Ethics oversight	Ethics Committee of Daping Hospital, Army Medical University (NO. 2020143)<br>Trial Registration: ChiCTR2100046643

Note that full information on the approval of the study protocol must also be provided in the manuscript.

Flow Cytometry

Plots

Confirm that:

☒ The axis labels state the marker and fluorochrome used (e.g. CD4-FITC).

☒ The axis scales are clearly visible. Include numbers along axes only for bottom left plot of group (a 'group' is an analysis of identical markers).

☒ All plots are contour plots with outliers or pseudocolor plots.

☒ A numerical value for number of cells or percentage (with statistics) is provided.

Methodology

Sample preparation	cells were collected by trypsinization at 48 h after treatment with IL-6, osimertinib, or both. Then, cells were washed three times with PBS, and resuspended at a density of 1 × 106 cells/mL. Next, cells were double-stained with PI for 10 min at ambient temperature in the dark using FITC Annexin V Apoptosis Detection Kit I (Sigma, Germany)

Instrument	Beckman Coulter Navios, CA, USA
Software	CytExpert version 2.3
Cell population abundance	10, 000 cells were collected for each cell line gated on their FSC/SCC properties.
Gating strategy	FSC/SSC gates were based on previous knowledge of the position of cells in a population based on these parameters due to the cell size and granularity.

☒ Tick this box to confirm that a figure exemplifying the gating strategy is provided in the Supplementary Information.
